# Association between Metabolic Syndrome and Leukocytes: Systematic Review and Meta-Analysis

**DOI:** 10.3390/jcm12227044

**Published:** 2023-11-11

**Authors:** Elena Raya-Cano, Manuel Vaquero-Abellán, Rafael Molina-Luque, Guillermo Molina-Recio, José Miguel Guzmán-García, Rocío Jiménez-Mérida, Manuel Romero-Saldaña

**Affiliations:** 1 Nursing, Pharmacology and Physiotherapy Department, University of Cordoba, 14071 Córdoba, Spain; z82racae@uco.es (E.R.-C.); mvaquero@uco.es (M.V.-A.); en1moreg@uco.es (G.M.-R.); z92guzgj@uco.es (J.M.G.-G.);; 2 Associated Group GA 16 Lifestyles, Innovation and Health, Maimonides Institute for Biomedical Research of Córdoba, University of Cordoba, 14071 Córdoba, Spain; z92rosam@uco.es

**Keywords:** metabolic syndrome, leukocytes, white blood cells, biologic marker

## Abstract

**Background:** Metabolic syndrome (MetS) is a group of metabolic abnormalities characterised by central obesity, hypertension, dyslipidaemia, and dysregulation of blood glucose, which is associated with the risk of diabetes, cardiovascular disease, and overall mortality. White blood cell count is a selective marker of acute infection and inflammation, which could provide information on the metabolic status of subjects. This study aims to provide the best evidence on the association between MetS and white blood cell count by determining the effect size of this biomarker. **Methods:** A systematic review and meta-analysis of studies indexed in the PubMed and Scopus databases were performed. Methodological quality was assessed using the STROBE tool, overall risk of bias using RevMan (Cochrane Collaboration), and quality of evidence using Grade Pro. **Results:** We included 14 articles comparing leukocyte concentrations in 21,005 subjects with MetS and 66,339 controls. Subjects with MetS had a higher mean leukocyte count, 0.64 cells ×10^9^/L; CI95% 0.55–0.72; *p* < 0.00001; I^2^ = 93%. **Conclusions:** An in-depth evaluation of the relationship of leukocytes in the pathophysiological process of MetS could lead to new insights into early diagnosis.

## 1. Introduction

Metabolic syndrome (MetS) is a group of metabolic abnormalities that includes central obesity, hypertension, dyslipidaemia, and blood glucose disorders. This condition is associated with an increased risk of developing diabetes, cardiovascular disease, and a raised overall mortality rate [1]. In addition, the incidence and prevalence of MetS have increased globally, making this non-communicable disease a major public health hazard [2,3]. Therefore, early diagnosis and prevention of MetS are essential. The underlying pathophysiology involves insulin resistance (IR), chronic low-grade inflammation, and oxidative stress, playing a crucial role in the pathogenesis of MetS [4,5].

Inflammatory markers are generally increased in patients with MetS, but the link between inflammation and the development of MetS is less well established. However, evidence suggests that changes in haematological parameters related to inflammatory processes, such as white blood cell count (WBC) and prothrombotic markers, may be associated with MetS [6,7]. WBC, neutrophils, and lymphocytes are common, inexpensive, and widely used markers of inflammation in the clinical setting [8]. These markers activate the main cell types involved in acute and chronic inflammation [9]. Additionally, white blood cells altered by chronic inflammatory risk factors are more likely to bind and adhere to vascular endothelium, which can cause capillary leukocytosis and eventually lead to vasoconstriction and hypertension [10].

Likewise, WBC count is directly associated with insulin resistance and, inversely, with insulin secretion. Concerning this, WBC count has been shown to predict both worsening insulin sensitivity and the incidence of type 2 diabetes [11]. Furthermore, due to hypertrophy-induced inflammation and leukocyte infiltration, adipose tissue loses sensitivity to insulin, resulting in increased lipolysis and impaired lipid storage, augmenting its dysfunctionality. As a result, free fatty acids and triglycerides are mobilised into the circulation, accumulating lipid derivatives in skeletal muscle, liver, and pancreatic B-cells, leading to impaired tissue function and systemic insulin resistance [12].

Thus, increased WBC may be directly involved in the pathogenesis of MetS by increasing the movement of inflammatory cells into adipose tissue. Prolonged maintenance or worsening of this metabolically dysfunctional state further perpetuates dysregulation of lipid metabolism and immune responses, increasing the individual’s risk of developing a wide range of chronic diseases [13,14].

In addition, previous studies have shown a significant relationship between WBC and MetS [6,15]. In this regard, it has been observed that the number of immune cell subtypes, specifically, the total number of leukocytes, lymphocytes, and monocytes, is higher in individuals with MetS [16]. Therefore, since chronic subclinical inflammation is implicated in the genesis of MetS and WBC can be used as a marker of inflammation, assessing the association between WBC count and the development of MetS may generate a new parameter to aid in its detection.

The primary aim of this systematic review and meta-analysis is to offer the most robust evidence regarding the correlation between Metabolic Syndrome (MetS) and leukocyte levels, ascertaining the magnitude of this biomarker’s impact.

## 2. Materials and Methods

### 2.1. Search Strategy and Eligibility Criteria

This systematic review and meta-analysis were conducted according to the criteria established by the PRISMA statement [17] (Supplementary Materials). The search was performed in the PubMed and Scopus databases, covering January 2017 to January 2022. The search methodology was formulated by amalgamating the following Medical Subject Headings (MeSH) descriptors: “metabolic syndrome”, “leukocytes”, and “white blood cells” with the Boolean operator AND. Cross-sectional and longitudinal studies investigating the association between MetS and leukocytes or articles collecting data related to both parameters were included. In addition, the results had to include the mean and standard deviation. Only manuscripts in English and Spanish and those collecting data on subjects older than 18 years were considered. Papers from subjects previously diagnosed with diabetes, obesity or active infections that could increase the level of leukocytes in their study groups were excluded. The systematic review was registered in PROSPERO with ID CRD42022228327.

### 2.2. Selection of Papers

E.R.C. and M.R.S. conducted independent reviews of all the articles retrieved in the search to remove duplicates. Subsequently, R.J.M., R.M.L., J.M.G.G., and G.M.R., four other authors, individually examined the titles and abstracts, applying eligibility criteria to select the articles that ultimately made it into the review. Lastly, M.V.A., the fifth author, served as a judge in the event of any discrepancies.

### 2.3. Data Extraction

One researcher (E.R.C) extracted the data, verified by a second investigator (R.J.M). A third researcher (M.R.S) decided in case of disagreement between them. Cohen’s Kappa index was used to assess the degree of agreement. We collected the following information from each study: citation, characteristics of the study population (including age and gender), study methodology, duration of follow-up, sample size, as well as the average and standard deviation of leukocyte levels in individuals with Metabolic Syndrome (MetS+) and those without Metabolic Syndrome (MetS−). In addition, the mean and standard deviation were extracted for reports collecting neutrophil, lymphocyte, and monocyte data. 

### 2.4. Evaluation of the Qualitative Synthesis

A team of four authors (R.M.L, R.J.M, E.R.C, and G.M.R) conducted a thorough qualitative synthesis assessment through a triple analysis:

(a) Methodological quality evaluation was performed using the STROBE (Strengthening the Reporting of Observational Studies in Epidemiology) statement [18] for observational studies.
(b) Risk of bias evaluation was conducted using the Cochrane Collaboration tool [19] integrated into the REVMAN 5.4.2 software (Cochrane Collaboration, Copenhagen, Denmark). This analysis assessed risks related to selection, conduct, detection, attrition, and reporting.
(c) Evaluating the evidence quality. Utilizing the Grade Pro tool (McMaster University and Evidence Prime), we constructed the evidence profile table, assigning specific levels as outlined [20]:
  - High: Strong assurance in aligning the actual and estimated effect;
  - Moderate: Reasonable confidence in the estimated effect. The actual effect may differ significantly;
  - Low: Restricted confidence in the estimated effect. The actual effect may deviate substantially from the estimate;
  - Very Low: Minimal confidence in the estimated effect. The actual effect is highly likely to vary extensively from the estimate.

### 2.5. Statistical Analysis (Evaluation of Quantitative Synthesis or Meta-Analysis)

The statistical computations and generation of forest and funnel plots for the meta-analysis were conducted using the Cochrane Review Manager software (RevMan 5.4.2). Given the variation in effect sizes among the included studies, a meta-analysis was executed utilizing the Mantel–Haenszel random-effects approach, following the DerSimonian and Laird model. The difference between arithmetic means with a 95% confidence interval was used to measure effect size. Leukocyte count was measured in cells ×10^9^/L. Publication bias risk was evaluated through an examination of the funnel plot. Heterogeneity was assessed by computing the Chi-square test and the inconsistency index (I^2^). Following the Cochrane Collaboration tool, heterogeneity was categorized as follows: unimportant (0–40%), moderate (30–60%), substantial (50–90%), and considerable (75–100%).

## 3. Results

### 3.1. Characteristics of the Studies

**Figure 1:**
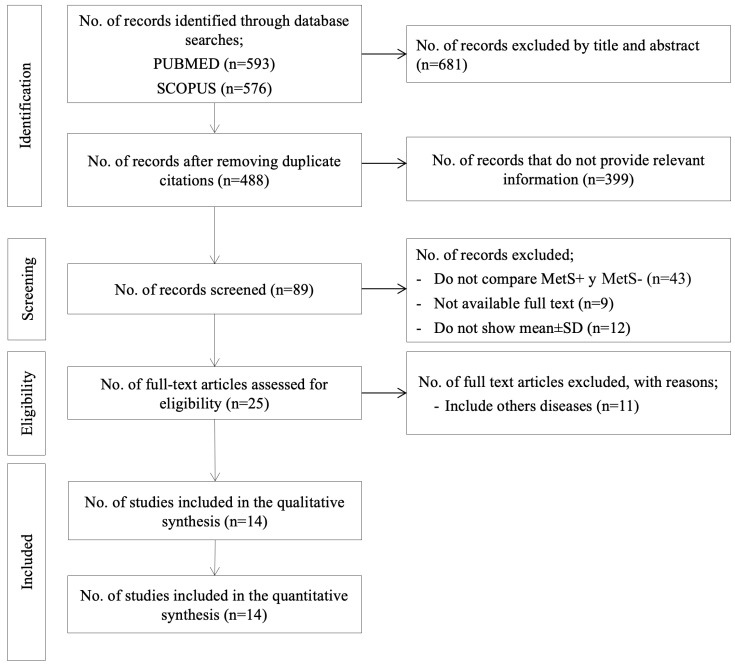
PRISMA flow chart. MetS, metabolic syndrome; SD, standard deviation.

The search yielded 89 records, of which 25 were identified for full-text review (Figure 1). Of these, 14 met the inclusion criteria and were therefore selected for systematic review and meta-analysis.

Regarding the research design, all studies were observational: 10 cross-sectional studies [10,21,22,23,24,25,26,27,28,29], 3 cohort studies [9,30,31], and 1 case–control study [32]. In total, the 14 papers compared leukocyte concentrations between 21,005 MetS+ and 66,339 MetS− subjects. The ages of the participants ranged from 18 to 85 years. Most of the papers (57.14%) [9,22,24,26,27,28,30,32] included participants of both sexes, but analysed the data globally; 3 studies (21.4%) included only men [21,23,25], and 3 others collected data from men and women separately [10,29,31]. In relation to provenance, half of the articles found were developed in the Chinese population [9,10,22,26,28,29,30,31]. In addition, neutrophil data were extracted from 7 articles [9,22,26,27,28,29,30], lymphocyte data from 6 studies [9,22,24,27,31,32], monocyte data from 4 papers [28,29,30,32], and eosinophil and basophil data from 2 manuscripts [28,29].

MetS was defined according to the National Cholesterol Education Program (NCEP-ATP III) third report criteria [33] in 7 research studies [22,23,24,27,29,31,32]; 3 studies [10,21,28] assessed MetS using the International Diabetes Federation (IDF) definition [34]; 2 studies [25,26] used harmonised criteria [35]; and 2 articles [9,30] as defined by the Chinese Diabetes Society [36].

The in-depth features of the chosen studies can be found in Table 1.

### 3.2. Methodological Quality Assessment

Every report scored 19 or higher out of the 22 items outlined in the STROBE reporting guidelines [18], placing them in the highest tercile. No articles were excluded for poor methodological quality. In Table 1, you can observe the individual scores assigned to each paper.

### 3.3. Bias Risk Analysis

**Figure 2:**
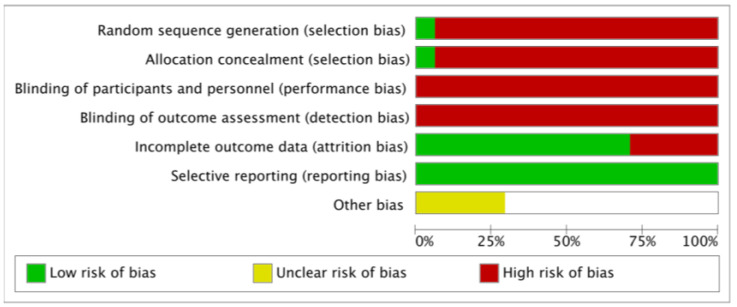
Overall risk of bias observed in the studies.

**Figure 3:**
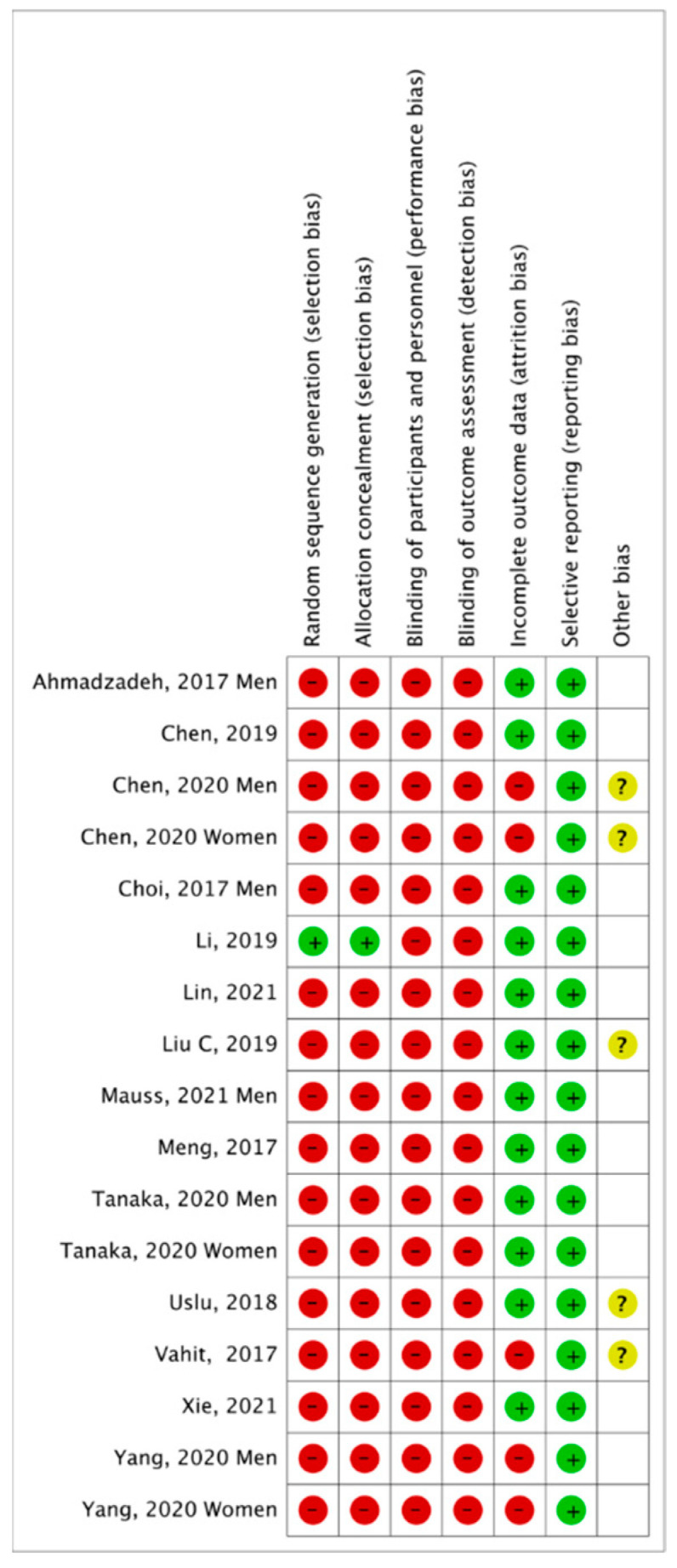
Summary of risk of bias by study [9,10,21,22,23,24,25,26,27,28,29,30,31,32].

Overall (Figure 2), it can be seen that the main biases were random sequential generation, allocation concealment, blinding of participants and personnel, and blinding of outcome assessment. Only one of the included articles collected data randomly with allocation concealment [30]. Figure 3 represents the individual assessment of the included studies.

### 3.4. Quantitative Analysis and Meta-Analysis

**Figure 4:**
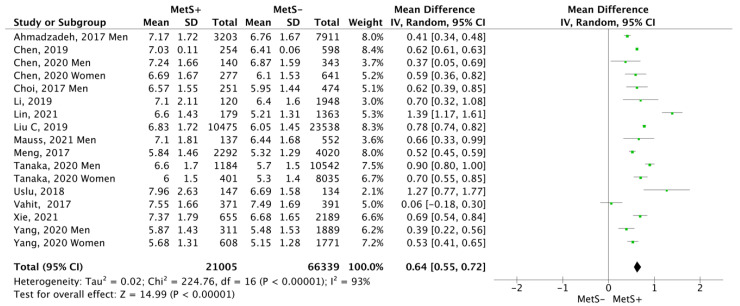
Results and summary statistics of studies analysing leukocyte levels in the total population with and without metabolic syndrome (MetS) [9,10,21,22,23,24,25,26,27,28,29,30,31,32].

Figure 4 shows the Forest Plot, including the results for both sexes from the 14 review articles. MetS+ subjects showed a higher mean leukocyte count, namely the mean difference was 0.64 cells ×10^9^/L (CI95% 0.55–0.72; *p* < 0.00001; I^2^ = 93%), compared to MetS− subjects.

**Figure 5:**
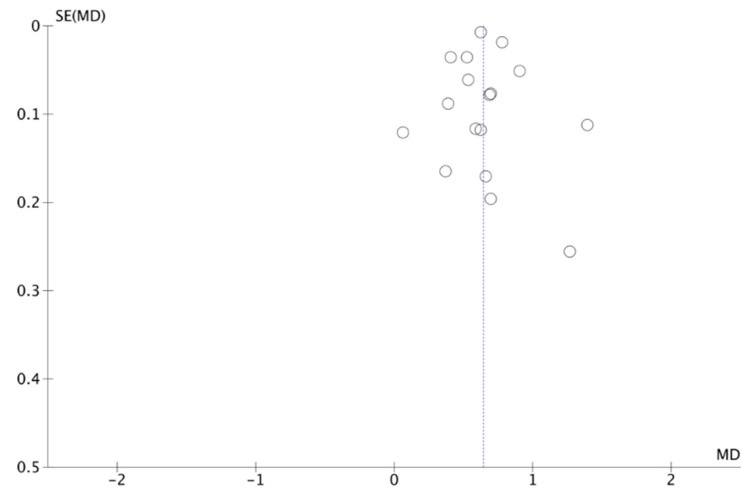
Funnel plot.

The Funnel Plot (Figure 5) shows a low risk of publication bias. The sensitivity analysis did not show that any study significantly affected the heterogeneity of the meta-analysis; therefore, no articles were excluded.

**Figure 6:**
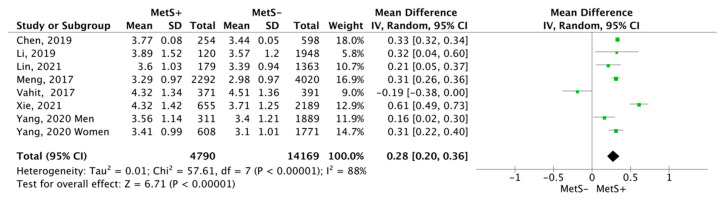
Results and summary statistics of studies analysing neutrophil levels in the total population with and without metabolic syndrome (MetS) [9,22,26,27,28,29,30].

MetS+ subjects showed a higher mean number of neutrophils, specifically, the mean difference was 0.28 cells ×10^9^/L (CI95% 0.2–0.36; *p* < 0.00001; I^2^ = 88%), compared to MetS− subjects (Figure 6).

**Figure 7:**
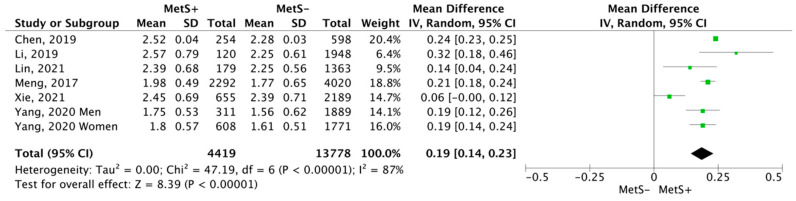
Results and summary statistics of studies analysing lymphocyte levels in the total population with and without metabolic syndrome (MetS) [9,22,26,28,29,30].

In relation to lymphocytes (Figure 7), MetS+ subjects showed a higher mean, the mean difference was 0.19 cells ×10^9^/L (CI95% 0.14–0.23; *p* < 0.00001; I^2^ = 87%), compared to MetS− subjects.

### 3.5. Quality of Evidence

Table 2 shows the evidence profile of the meta-analysis, providing specific information regarding the overall certainty of the evidence of the studies included in the comparison, the magnitude of the studies examined, and the sum of the data available for the outcomes assessed.

## 4. Discussion

A comprehensive review and meta-analysis were performed to examine the latest evidence regarding the association between Metabolic Syndrome (MetS) and leukocyte levels. Fourteen articles were selected to quantify the size effect and the limitations that have conditioned their results. All demonstrated sufficient reliability and methodological quality regarding the association between leukocytes and MetS.

The present meta-analysis shows the relationship between the level of leukocytes and MetS. The leucocyte concentration in the 21,005 MetS+ subjects was significantly higher than in the group of 66,339 controls (mean difference (MD): 0.64 cells ×10^9^/L; CI95% 0.55–0.72; *p* < 0.00001).

The results of this review support how elevated white blood cell count is closely related to MetS. The mechanisms that explain this association are not entirely clear, but some possibilities have been suggested. On the one hand, IR, defined as the decreased capability of insulin to stimulate glucose uptake by muscle and adipose tissues and to suppress hepatic glucose production [37], may contribute to metabolic disturbances and accumulation of inflammatory markers, such as total leukocytes and other inflammatory factors [29].

On the other hand, MetS indicates metabolic dysregulation or dysfunction, strongly associated with atherosclerotic cardiovascular disease and often accompanied by chronic low-grade inflammation [4,38]. This inflammation can induce the synthesis of several groups of cytokines and proteolytic enzymes and decrease the formation of prostacyclin and nitric oxide, which can cause impaired endothelial integrity and functional impairment, leading to an increase in white blood cells and their subtypes [9,11,13]. Furthermore, TNF-a has been shown to be consistently expressed in adipose tissue, and these proinflammatory cytokines lead to elevated leukocyte levels [39]. This increase may lead to hypertension and loss of vasodilatory capacity [40]. The study by Marques P et al. [41] reports that neutralising chemokine axes partially inhibit leukocyte adhesion through altered adhesiveness of proinflammatory monocytes to dysfunctional endothelium, suggesting a potential link between the systemic inflammatory response and the development of CVD in MetS.

In addition, Lorenzo et al. note that elevated total white blood cell, neutrophil, and lymphocyte counts can be detected in people at increased risk of diabetes due to insulin sensitivity and low-grade inflammation [14]. Metabolic alterations and inflammation enter a vicious cycle of T-cell activation, senescence, and proinflammatory cytokine production that worsens pathological conditions [42].

Our results are consistent with reported associations between leukocytes and MetS. Previous longitudinal and cross-sectional studies have associated WBC with the incidence and prevalence of MetS [6,43]. The cross-sectional study by Babio et al. [44] demonstrates that WBC count was associated with increased risk and prevalence of MetS and concluded that WBC count is positively associated with three parameters used as defining criteria for MetS: hyperglycaemia, HDL-cholesterol, and hypertriglyceridaemia. Therefore, circulating white blood cells could represent a critical factor in the study of obesity and its associated comorbidities, such as MetS and CVD [45]. In addition, the study by Wang et al. [46] confirms that monitoring longitudinal changes in leukocyte markers may help provide a strategy for primary prevention of future cardiovascular events. Thus, cardiometabolic risk factors contribute to developing and worsening this proinflammatory and prothrombotic state associated with MetS, leading to detrimental metabolic conditions. Many of these conditions are acquired through lifestyle and are modifiable, indicating the importance of prevention and treatment methods to improve cardiometabolic risk factors to reduce their impact on MetS [47,48].

## 5. Limitations and Strengths

In this kind of research, evaluating the potential biases in study methodologies is a crucial concern under PRISMA guidelines. Studies with similar methodologies but discrepancies in quality may have biased results. The quality of the evidence obtained is “very low” because observational studies have been analysed. These study designs pose a high bias risk and show a very high inconsistency (heterogeneity). The authors were unable to thoroughly examine the impact of adjustment for all known and potential risk factors due to the varying degrees of adjustment for confounding factors across individual studies. One of the main strengths of this review is a large sample size of subjects with and without MetS was included, which increased the study’s statistical power. However, analysing the findings in this systematic review and meta-analysis should be conducted with caution, considering some limitations. Firstly, non-randomised comparisons in observational studies may suffer from biases, which could affect the results and thus weaken the strength of the evidence. Secondly, the different criteria or definitions used to diagnose MetS in the included studies may influence the determination and identification of affected individuals. Also, the treatment approach and health objectives may change depending on the definition. Third, with increasing age, there is decreased adaptive immunity and increased inflammation or immunoaging, which affects the levels of proinflammatory cytokines that can alter the leukocyte profile [49]. Fourth, most studies come from the Far East region, making it difficult to generalize the results to other countries. Fifth, further research is required to identify the importance of increased neutrophils and lymphocytes in MetS and other cardiovascular diseases. Finally, another limitation was that no additional strategies were used in the current search to locate unpublished reviews (grey literature).

## 6. Conclusions

The results have shown that subjects with MetS have higher levels of leukocytes (0.64 cells ×10^9^/L; CI95% 0.55–0.72; *p* < 0.00001), neutrophils (0.28 cells ×10^9^/L; CI95% 0.2–0.36; *p* < 0.00001), and lymphocytes (0.19 cells ×10^9^/L; CI95% 0.14–0.23; *p* < 0.00001). These results provide a rationale for further evaluation of the relationship of leukocytes in the pathophysiological process of MetS. They could lead to new insights in early diagnosis, identification of new biomarkers, and discovery of new therapeutic targets for pharmacological interventions. Further research is therefore required to identify the importance of white blood cell counts in MetS or other cardiovascular diseases.

## Figures and Tables

**Table 1:** Characteristics of included studies (*n* = 14).

Author, Year, Country	Study Design	STROBE^18^ Reporting Guidelines	Age of Participants	No. of Subjects MetS+/MetS−	MetS Criteria	Results					
Ahmadzadeh et al., 2017, Iran [21]	Cross-sectional study	19	Men MetS+ 41.4 ± 9.9 MetS− 36.4 ± 9.6	Men 3203/7911 Total 11,114	IDF	Increased WBC (*p* < 0.001) is related to a higher number of MetS criteria. Men MetS+ 7.2 ± 1.7 (WBC) MetS− 6.7 ± 1.7 (WBC)					
Chen et al., 2019, China [22]	Cross-sectional study	20	MetS+ 56.5 ± 0.5 MetS− 47.6 ± 0.4	254/598 Total 852	NCEP ATP III	Elevated WBC levels in MetS+ subjects. MetS+ 7.03 ± 0.1 (WBC) MetS− 6.4 ± 0.06 (WBC)					
Chen et al., 2020, China [10]	Cross-sectional study	19	Women MetS+ 60.7 ± 10.0 MetS− 52.6 ± 12.7 Men MetS+ 57.2 ± 10.5 MetS− 54.8 ± 13.5	Women 277/641 Total 918 Men 140/343 Total 483	IDF	Haematological parameters, including WBC and subtypes, correlate with the occurrence of MetS.					
						Women MetS+ 6.69 ± 1.67 (WBC) MetS− 6.1 ± 1.53 (WBC)			Men MetS+ 7.24 ± 1.66 (WBC) MetS− 6.87 ± 1.59 (WBC)		
Hoi et al., 2017, Japan [23]	Cross-sectional study	21	Men MetS+ 49.5 ± 6.5 MetS− 48.8 ± 6.1	Men 251/474 Total 725	NCEP ATP III	Significantly higher white blood cell count in MetS+ subjects. Men MetS+ 6.57 ± 1.55 (WBC) MetS− 5.95 ± 1.44 (WBC)					
Li et al., 2019, China [30]	Retrospective cohort study	19	MetS+ 52.5 ± 13.6 MetS− 41.1 ± 13.3	120/1948 Total 2068	Chinese Diabetes Society	The MetS+ group had higher TSH and inflammation levels, indicated by higher WBC, LY, and Mo/HDL.					
						MetS+ 7.1 ± 2.11 (WBC) MetS− 6.4 ± 1.6 (WBC) MetS+ 2.57 ± 0.79 (Lymphocyte) MetS− 2.25 ± 0.61 (Lymphocyte)			MetS+ 3.89 ± 1.52 (Neutrophil) MetS− 3.57 ± 1.2 (Neutrophil) MetS+ 0.43 ± 0.15 (Monocyte) MetS− 0.39 ± 0.13 (Monocyte)		
Lin et al., 2021, China [9]	Cohort study	20	MetS+ 45 ± 11.6 MetS− 44.9 ± 13.18	179/1363 Total 1542	Chinese Diabetes Society	Subjects with MetS+ have higher levels of leukocytes, neutrophils, and total lymphocytes. Elevated levels of leukocytes, neutrophils, and lymphocytes increased the incidence of MetS.					
						MetS+ 6.6 ± 1.4 (WBC) MetS− 6.21 ± 1.3 (WBC)		MetS+ 3.6 ± 1.03 (Neutrophil) MetS− 3.39 ± 0.94 (Neutrophil)		MetS+ 2.39 ± 0.68 (Lymphocyte) MetS− 2.25 ± 0.56 (Lymphocyte)	
Liu C et al., 2019, Taiwan [24]	Cross-sectional study.	19	MetS+ 50.4 ± 11.1 MetS− 45.6 ± 11.1	10,475/23,538 Total 34,013	NCEP ATP III	Inflammatory biomarkers (WBC, CRP, and Hs-CRP), lipid markers (total cholesterol, triglycerides, and LDL-cholesterol), and glycaemic markers (fasting glucose, HbA1c, insulin, HOMA-IR, and SUA) were on average higher in the MetS+ group than in MetS− (*p* < 0.001). MetS+ 6.83 ± 1.72 (WBC) MetS− 6.05 ± 1.45 (WBC)					
Mauss et al., 2020, Germany [25]	Cross-sectional study	19	Men MetS+ 49.5 ± 8.1 MetS− 44.5 ± 9.9	Men 137/552 Total 689	Harmonised criteria	Total leukocyte count and CRP were higher in the MetS+ group, while leukocyte ratios showed no significant differences. Men MetS+ 7.1 ± 1.81 (WBC) MetS− 6.44 ± 1.68 (WBC)					
Meng et al., 2017, China [26]	Cross-sectional study	21	MetS+ 52.7 ± 9.7 MetS− 48.9 ± 9.7	2292/4020 Total 6312	Harmonised criteria	They observe that leukocyte, neutrophil, and lymphocyte concentrations are associated with MetS.					
						MetS+ 5.84 ± 1.46 (WBC) MetS− 5.32 ± 1.29 (WBC)		MetS+ 3.29 ± 0.97 (Neutrophil) MetS− 2.98 ± 0.97 (Neutrophil)		MetS+ 1.98 ± 0.49 (Lymphocyte) MetS− 1.77 ± 0.65 (Lymphocyte)	
Tanaka et al., 2020, China [31]	Cohort study	19	Women MetS+ 55.2 ± 10.4 MetS− 44.8 ± 9.8 Men MetS+ 50.3 ± 9.4 MetS− 44.8 ± 9.7	Women 401/8035 Total 8436 Men 1184/10,542 Total 11,726	NCEP ATP III	Higher levels of WBC are observed in the MetS group.					
						**Women** MetS+ 6.0 ± 1.5 (WBC) MetS− 5.3 ± 1.4 (WBC)			**Men** MetS+ 6.6 ± 1.7 (WBC) MetS− 5.7 ± 1.5 (WBC)		
Uslu et al., 2018, Turkey [32]	Case–control study	19	MetS+ 47 ± 13.5 MetS− 44 ± 15.2	147/134 Total 281	NCEP ATP III	MHR is a useful inflammatory marker to assess MetS and disease severity.					
						MetS+ 7.96 ± 2.63 (WBC) MetS− 6.69 ± 1.58 (WBC)			MetS+ 0.59 ± 0.26 (Monocyte) MetS− 0.48 ± 0.16 (Monocyte)		
Vahit et al., 2017, Turkey [27]	Cross-sectional study	20	MetS + 57.4 ± 8.8 MetS− 56.3 ± 9.1	371/391 Total 762	NCEP ATP III	MRLs such as MHR may be novel and valuable indicators in MetS.					
						MetS+ 7.55 ± 1.66 (WBC) MetS− 7.49 ± 1.69 (WBC)			MetS + 4.32 ± 1.34 (Neutrophil) MetS− 4.51± 1.36 (Neutrophil)		
Xie et al., 2021, China. [28]	Cross-sectional study	19	MetS+ 26.1 MetS− 25.7	655/2189 Total 2844	IDF	Lasso’s logistic regression algorithm helped to identify MetS with high accuracy in an occupational population.					
						MetS+ 7.37 ± 1.79 (WBC) MetS− 6.68 ± 1.65 (WBC) MetS+ 0.42 ± 0.15 (Monocyte) MetS− 0.39 ± 0.13 (Monocyte) MetS+ 0.17 ± 0.13 (Eosinophil) MetS− 0.18 ± 0.18 (Eosinophil)			MetS+ 2.45 ± 0.69 (Lymphocytes) MetS− 2.39 ± 0.71 (Lymphocytes) MetS+ 4.32 ± 1.42 (Neutrophil) MetS− 3.71 ± 1.25 (Neutrophil) MetS+ 0.07 ± 0.16 (Basophil) MetS− 0.05 ± 0.11 (Basophil)		
Yang et al., 2020, China. [29]	Cross-sectional study	19	≥60 years	Women 608/1771 Total 2379 Men 311/1889 Total 2200	NCEP ATP III	They observe interactions between leukocytes, monocytes, neutrophils, and sex in MetS.					
						**Women** MetS+ 5.68 ± 1.31 (WBC) MetS− 5.15 ± 1.28 (WBC) MetS+ 1.8 ± 0.57 (Lymphocytes) MetS− 1.61 ± 0.51 (Lymphocytes) MetS+ 0.3 ± 0.1 (Monocyte) MetS− 0.28 ± 0.1 (Monocyte) MetS+ 3.41 ± 0.99 (Neutrophil) MetS− 3.1 ± 1.01 (Neutrophil) MetS+ 0.13 ± 0.11 (Eosinophil) MetS− 0.13 ± 0.13 (Eosinophil) MetS+ 0.03 ± 0.02 (Basophil) MetS− 0.03 ± 0.02 (Basophil)			**Men** MetS+ 5.87 ± 1.43 (WBC) MetS− 5.48 ± 1.53 (WBC) MetS+ 1.75 ± 0.53 (Lymphocytes) MetS− 1.56 ± 0.62 (Lymphocytes) MetS+ 0.35 ± 0.16 (Monocyte) MetS− 0.34 ± 0.13 (Monocyte) MetS+ 3.56 ± 1.14 (Neutrophil) MetS− 3.4 ± 1.21 (Neutrophil) MetS+ 0.16 ± 0.15 (Eosinophil) MetS− 0.14 ± 0.14 (Eosinophil) MetS+ 0.04 ± 0.02 (Basophil) MetS− 0.03 ± 0.02 (Basophil)		

CRP, C-reactive protein; HbA1c, haemoglobin A1c; HOMA-IR, Homeostatic Model Assessment of Insulin Resistance; hsCRP, high-sensitivity C-reactive protein; IDF, International Diabetes Federation; LY, lymphocytes; LMR, lymphocyte-to-monocyte ratio, MetS, metabolic syndrome; MHR, monocyte to high-density lipoprotein cholesterol ratio; Mo/HDL, monocyte/high-density lipoprotein; NCEP ATP III, National Cholesterol Education Program Adult Treatment Panel III; STROBE, Strengthening the Reporting of Observational Studies in Epidemiology; SUA, serum uric acid; TSH, thyroid-stimulating hormone; WBC, white blood cells.

**Table 2:** Evidence profile with GRADE pro for the meta-analyses.

Certainty Assessment							No. of Subjects		Size of the Effect	Quality of Evidence
No. of Studies	Study Design	Risk of Bias	Inconsistency	Indirect Evidence	Imprecision	Other Considerations	MetS+	MetS−	Mean Difference (95% CI)	
Meta-analysis White blood cells										
*n* = 14	Observational studies	serious	Very serious	It is not serious	It is not serious	dose-response gradient	21,005	66,339	0.64 (0.55–0.72)	⨁◯◯◯ Very low
Meta-analysis Neutrophils										
*n* = 7	Observational studies	serious	Very serious	It is not serious	It is not serious	dose-response gradient	4790	14,169	0.28 (0.2–0.36)	⨁◯◯◯ Very low
Meta-analysis Lymphocytes										
*n* = 6	Observational studies	serious	Very serious	It is not serious	It is not serious	dose-response gradient	4419	13,778	0.19 (0.14–0.23)	⨁◯◯◯ Very low

MetS, metabolic syndrome; CI, confidence interval.

## Data Availability

All data generated or analysed during this study are included in this published article and its Supplementary Materials. The primary findings of the study are incorporated in the article; for additional information, please contact the corresponding author.

## Supplementary Materials

The following supporting information can be downloaded at: https://www.mdpi.com/article/10.3390/jcm12227044/s1, PRISMA 2020 Checklist [50].
